# Fluorescent Nanoparticles for the Guided Surgery of Ovarian Peritoneal Carcinomatosis

**DOI:** 10.3390/nano8080572

**Published:** 2018-07-26

**Authors:** Tristan Mangeolle, Ilya Yakavets, Sophie Marchal, Manon Debayle, Thomas Pons, Lina Bezdetnaya, Frédéric Marchal

**Affiliations:** 1 Centre de Recherche en Automatique de Nancy, Centre National de la Recherche Scientifique UMR 7039, Université de Lorraine, Campus Sciences, Boulevard des Aiguillette, 54506 Vandoeuvre-lès-Nancy, France; t.mangeolle@nancy.unicancer.fr (T.M.); i.yakavets@nancy.unicancer.fr (I.Y.); s.marchal@nancy.unicancer.fr (S.M.); l.bolotine@nancy.unicancer.fr (L.B.); 2 Research Department, Institut de Cancérologie de Lorraine, 6 avenue de Bourgogne, 54519 Vandoeuvre-lès-Nancy, France; 3 Laboratory of Biophysics and Biotechnology, Belarusian State University, 4 Nezavisimosti Avenue, 220030 Minsk, Belarus; 4 LPEM, ESPCI Paris, PSL Research University, CNRS, Sorbonne Université, 75005 Paris, France; manon.debayle@espci.fr (M.D.); thomas.pons@espci.fr (T.P.); 5 Surgical Department, Institut de Cancérologie de Lorraine, 6 avenue de Bourgogne, 54519 Vandoeuvre-lès-Nancy, France

**Keywords:** cancer imaging, cytoreduction surgery, fluorescent nanoparticle, near-infrared, short-wave infrared

## Abstract

Complete surgical resection is the ideal cure for ovarian peritoneal carcinomatosis, but remains challenging. Fluorescent guided surgery can be a promising approach for precise cytoreduction when appropriate fluorophore is used. In the presence paper, we review already developed near- and short-wave infrared fluorescent nanoparticles, which are currently under investigation for peritoneal carcinomatosis fluorescence imaging. We also highlight the main ways to improve the safety of nanoparticles, for fulfilling prerequisites of clinical application.

## 1. Introduction

### 1.1. Epidemiology

Some peritoneal and gastrointestinal malignancies show preferential dissemination and invasion into peritoneal cavity, leading to a peritoneal carcinomatosis with substantial consequences on survival [1]. Among these malignancies, Epithelial Ovarian cancers (EOC) remain the fifth leading cause of death with a five-year survival rate of only 46%, albeit EOCs are only the 8th most common cancer in women [2]. The poor prognosis of these cancers is mainly due to the absence of specific early symptoms, leading to late diagnosis [3]. When confined to the ovary or the regional lymph nodes, EOC provides respectively 92.5 and 73% of survival at five years but they only represent 15 and 20% of newly diagnosed EOC respectively. 65% of EOC are diagnosed at distant stage with a survival rate of 28.9% [4].

Distant stages are characterized by the presence of cancer cells in the peritoneal cavity and/or in the retroperitoneal lymph nodes, where they can induce peritoneal carcinomatosis [5]. Peritoneal carcinomatosis suggests metastases, which in turn are localized on the peritoneum and the peritoneal organs, varying in size from microscopic lesions to cancerous masses of several centimeters [6]. Ultimately, peritoneal carcinomatosis progression leads to debilitating ascites and, above all, intestinal obstruction and subsequent lethal outcomes [7].

### 1.2. Conventional Treatment

The frontline treatment for peritoneal carcinomatosis of ovarian origin associates extensive surgery with peri-operative chemotherapy, mainly by paclitaxel and cisplatin, to remove the whole cancerous mass.

The main objective of extensive surgery is to excise macroscopic cancerous implants from the ovary and from the entire peritoneal cavity. Initially considered as palliative treatment to alleviate abdominal pain, extensive surgery was progressively developed for a curative intent with total removal of cancerous lesions [8], and was finally standardized by Sugarbaker [9]. However, despite many improvements, this procedure remains challenging.

First, surgeons can rely only on pre-operative imaging to distinguish all cancerous lesions, mainly by position emission tomography (PET), computed tomography (CT) or magnetic resonance imaging (MRI) [10], eventually combined with ultrasound, Doppler and laparoscopic observation [11].

During surgery, surgeons must explore the whole peritoneal cavity, delineated by a serous membrane (the peritoneum), with organs such as liver, spleen, pancreas, and the whole gastrointestinal tract. Altogether, peritoneum and peritoneal organs represent an area almost equivalent to that of the body [12]. Exploration of this huge surface requires many hours and can be achieved only by experienced surgeons. The goal of primary surgery is a complete resection, without any residual disease [13]. To eliminate residual cancerous cells, several cycles of intravenous platinum-based chemotherapy combined with paclitaxel is performed [14].

To treat residual microscopic metastases and thus to achieve a complete cytoreduction initiated with the surgical procedure, it is necessary to increase the local drug concentration by intraperitoneal injections [15]. It was quickly shown that the peritoneal membrane limits the plasmatic passage in case of local injection of ionized and lipid insoluble compounds [16,17]. Therefore, hydrophilic drugs injected by intraperitoneal are maintained at higher concentrations than after intravenous injection, with a lower risk of systemic toxicity. 

Although this approach was clinically validated [18,19,20], one limiting factor consisting of a shallow drug penetration in the tumor (no more than few millimeters) considerably reduced its clinical efficacy on gross residual colon tumors [21]. Recent studies show improved survival rate for ovarian cancer patients treated by intraperitoneal chemotherapy, with better and longer survival rate [22,23]. Combination of hyperthermy and intraperitoneal chemotherapy was recently confirmed for ovarian cancer treatment [24], showing improved survival without higher rates of side effects [25].

Irrespective of chemotherapy modalities, the residual disease after surgery remains one of the primary prognosis factors [26,27,28]. Survival at five years is closely related to the absence (60% of totally debulked patients) or the presence of microscopic metastases (30% for patient with “optimal” (<1 cm) residual disease) [29]. Moreover, despite complete surgery, early post-operative computerized tomography detects sub-optimal (>1 cm) residual tumors in almost half of the patients [30].

## 2. Fluorescence Guided Surgery

While chemotherapy has undergone adjustments and its optimization by hyperthermia is still debated, surgical debulking still depends on the extensive experience of the surgeon and his/her own ability to detect tumor deposits in the peritoneal cavity [31]. Attempts have been made to search for complementary solutions to enhance surgeon guidance. Among other options, fluorescence guided surgery (FGS) is highly demanded, especially in oncological surgery [32].

Tissue offers various autofluorescence patterns under ultraviolet illumination. Therefore, ultraviolet illumination was tested to detect cancerous tissue in the middle of the 20th century with some success [33]. Moore improved the technique by using the difference of retention between cancerous and healthy tissue of intravenous injected fluorescent dye, the fluorescein [34]. This technique was further applied with success to guide cerebral tumor resection [35]. Many improvements have been introduced since that time to FGS with fluorescein for glioblastoma surgical treatment and a similar approach to detect ovarian peritoneal carcinomatosis generated substantial improvements [36]. Van Dame and co-workers used as a target the predominant folate receptor sur-expression in ovarian cancer cells, combining fluorescein isothiocyanate (FITC) and folate. By means of filters and a fluorescence-specific camera, they increased the detection rate of residual disease four-fold [37]. The development of high-resolution cameras considerably contributed to real-time imaging of cancerous tissue with effective contrast and improved information accessible to the surgeon [32]. Another advantage provided by cameras was the possibility to use near-infrared (NIR) fluorescent dyes, invisible to human eye. Visible fluorescent dyes (fluorescein for example) are detectable mainly on the surface of tissue, no deeper than few millimeters, due to the absorption of biological chromophores (i.e., melanin, fat, hemoglobin, etc.). 

By contrast, NIR fluorescence is weakly absorbed by the tissues, allowing a deeper detection (up to five millimeters) [38] and even a whole-body fluorescence imaging for small animals such as rodents [39]. From that point of view, FGS benefited from extensive development and application of NIR-fluorescent dyes [40,41,42]. Among other available NIR-fluorescent dyes, indocyanine green (ICG) is currently the spearhead of the probes applied for FGS purpose. This dye, developed in the middle of the 20th century, is one of the few Food and drug administration (FDA)-approved NIR dyes [43]. ICG was indicated in patients for the measurements of cardiac output, liver function, blood flow and retinal angiography, as well as tolerated and hepatic cleared dye. It has also been tested for sentinel lymph node mapping and cancer imaging [44]. Even though ICG has no specificity for cancer cells, its high affinity for plasma proteins results in a preferential accumulation of ICG-protein complex in the tumor vasculature. Tumor anarchic vasculature offers larger lumen and fenestration, facilitating both the permeability and retention of macromolecules, known as Enhanced Permeability and Retention (EPR) effect [45]. The only known exception is the hepato-cellular carcinoma, which displays specificity for ICG, probably because of their hepatic cell remnant characteristics [46].

In the case of ovarian cancer, encouraging results were obtained with intravenously injected ICG in mice, allowing the detection of few millimeters of peritoneal metastases from different origins [47]. However, the first clinical results were contradictory: high sensitivity was associated with low specificity, with a high rate (62%) of false positive non-malignant lesions being observed [48]. Another problem, raised by the hepatic clearance of ICG, was the fluorescent contamination of the gastrointestinal tract that hampers tumor implant detection [49].

From these observations, the authors identified the urgent need for targeting probes rather than passive probes. With this aim, OTL-38, the NIR folate-targeted counterpart of the FITC-folate probe, was clinically tested, showing encouraging results of a higher signal-to-background ratio (SBR) [50]. As expected, OTL-38 allows deeper tumor detection (almost one centimeter below tissue surface) than with FITC-folate. However, irrespective of the NIR-fluorescent dyes used, light excitation and emission scattering limit the detection depth to a few millimeters and the surgeon still needs a pre-operative CT/MRI scan or other intraoperative imagery modalities for precise and exhaustive tumor localization and surgery planning [38]. Thus, for ovarian peritoneal carcinomatosis and peritoneal malignancies, the ideal probe for FGS should be multimodal by associating NIR dye, targeting moiety and another imaging agent for either CT or MRI to overcome the lack of specificity and limitation of fluorescence depth of detection. 

Until now, only one chemical multimodal probe has reached the phase I clinical trial for renal carcinoma. This probe consists in an antibody (girentuximab) directed against the carbonic anhydrase IX (CAIX), a common target of renal cancerous cells, bound to infrared (IR) fluorescent dye CW800 and the radioactive indium isotope ^111^In. Early results showed better fluorescent detection of CAIX-positive tumors by using pre-operative SPECT/CT imaging and intraoperative gamma camera. However, the authors noted that the fluorescence intensity had been attenuated by the surrounding fibrous tissue and the tumor capsule [51]. Irrespective of such chemical construction of the probe and the real benefit of multimodal imaging, the intrinsic low photostability and fluorescence shared by most chemical fluorescent dyes raise at least three challenging problems for FGS application.

First, the low photostability of the chemical dye implicates either limited time for surgery, which is not recommended for achieving total peritoneal cytoreduction, or higher amount of injected dye, which seems hazardous because of the dye toxicity. Second, relatively poor fluorescence emitted by the dye decreases the contrast between labeled and unlabeled tissue. To quantify the contrast, SBR is used. SBR measures the sensitivity of imaging device, and remains “the key determinant of sensitivity, detectability, and linearity in optical imaging” [52]. The lack of brightness and weak photostability of organic dyes reduce the SBR to a value above 2 [49], while the reference research in imaging establishes that SBR must be above 5 to reliably identify the object with absolute certainty [53]. Finally, this kind of “chemical fluorophore-based” construction is obviously difficult to adapt to another probe. Third, similar to ICG, CW800 is slowly excreted through the hepatobiliary way [49], resulting in contamination of the surgical field by the remnant unbounded fluorescent dye [54].

To summarize this part of the review on clinical advances in FGS, it is obvious that future probes will require bright and photo-resistant IR fluorescent dyes adaptable to multimodality and tumor targeting. In addition, the probe must be safe and rapidly excreted from the body to avoid fluorescence “contamination” and risk of toxicity in the long term. NIR nanoparticles (NPs) constitute alternative and seductive chemical constructs with the potential to fulfill all these requirements.

## 3. Overview of NIR Nanoparticles

**Figure 1:**
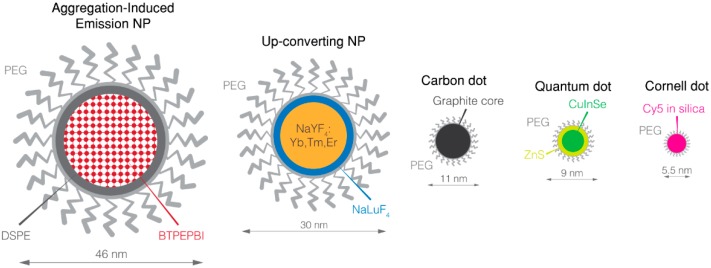
Overview of NIR nanoparticles. BTPEPBI: 1,7-tetraphenylethene modified 3,4:9,10-Tetracarboxylic perylene bisimide; Cy5: cyanine 5; DSPE: 1,2-distearoyl-*sn*-glycero-3-phosphoethanolamine-*N*-[amino(polyethylene glycol)]; NP: nanoparticle; PEG: poly(ethyleneglycol).

NIR-fluorescent NPs (Figure 1) possess common advantages (Table 1). First, they have higher brightness, which is the product of a far superior molar attenuation coefficient (absorption of light per mol) and very satisfying quantum yields (the ratio between emitted and absorbed photons) than any organic fluorophores, providing higher SBR [55]. In the case of long operative time such as during cytoreduction, NIR NPs maintain photostability without the production of toxic photoproducts. 

Similar to protein/ICG complexes, NPs can accumulate in tumors by EPR effect. Finally, most of them offer a versatile surface which can be easily modified for targeting and combined with another imaging modality to achieve even more effective multimodal probe.

### 3.1. Quantum Dots

Quantum dots (QD) are small fluorescent nanocrystals composed of semiconductor compounds. Unlike organic fluorophores, QD offer broad absorption and narrow emission spectra. Their emission wavelength depends on the composition and the size of nanocrystal (e.g., 3 nm PbS QD emits around 800 nm while the increasing diameter up to 6.5 nm leads to the emission wavelength more than 1500 nm) [66].

They are mainly synthetized through colloidal chemical syntheses, under inert atmosphere, where metallic precursors are suspended in organic solvent, such as octadecene, and heated at high temperature. The precursors decompose to form monomers that nucleate, thus creating very small nanocrystals. The second step is the growing stage of the nuclei and QD increase in size until they reach the desired one. Then, the solution is cooled very quickly to stop the growth. Subsequently, hydrophobic QD are transferred into water through ligand exchange (using mercaptopropionic acid for instance) or phospholipid micelle encapsulation. These syntheses allow very high quantum yield, even in the near-infrared range. The synthesis of hydrophilic QD has also been developed through the hydrothermal process, where organic solvent is replaced by water with either stabilizer or reverse micelles. However, these last hydrothermal syntheses have a higher polydispersity and a lower quantum yield in comparison with organic synthesis. 

QD are currently among the brightest known NPs. In addition, they possess longer fluorescence lifetime, from tens to hundreds of nanoseconds or even microseconds, which may be used to selectively detect QD fluorescence while eliminating autofluorescence background [67]. Therefore, QDs with variable composition and size have been developed toward their clinical applications in FGS.

Beside these attractive optical properties, the weakness of most NIR QDs comes from their composition, which is commonly based on heavy metals, such as cadmium (Cd) or lead (Pb). Despite their in vitro stability, QDs can quickly degrade in the hepatocyte cells (Hep G2) model. Following intravenous injection, QDs typically accumulate mainly in the liver and the kidney with therefore hazardous long-term consequences [68,69]. Toxicity results from Cd accumulation in the liver while Te mainly accumulates in the kidney [69,70]. The ionic leach of Cd is presumed to be the main cause of QD toxicity since the production of induced oxidative stress was proven through the role of metallothionein in cadmium retention [71]. Therefore, to protect QD from degradation, a shell constituted by different compounds such as zinc sulfide (ZnS) was placed around the QD core [72]. In addition, NIR-emitting QDs based on less toxic components, such as silver (Ag) or Indium (In), have also been developed [73]. QDs can easily be combined with other imaging modality, such as paramagnetic ion to obtain multimodal probe [56], while functionalization of their surface chemistry enables grafting of targeting moieties [74].

### 3.2. Up-Converting Nanoparticles (UCNP)

Up-Converting Nanoparticles (UCNPs) represent another type of solvothermal or hydrothermal made fluorescent nanocrystals, based on lanthanide atoms. The mechanism of NIR fluorescence emitted by UCNP is particular. These NPs emit in the NIR after excitation at longer wavelengths (usually 980 nm), through a multiphoton conversion process [75,76]. In addition to providing excellent penetration depth, this modality eliminates the autofluorescence background [77]. NIR UCNPs have already been used for murine peritoneal carcinomatosis imaging, showing satisfying imaging properties. The authors highlighted the good biocompatibility of UCNPs and the fact that some lanthanides, such as chelate gadolinium, are already FDA-approved for MRI imaging [58], and seem to be less concerned by toxicity issues [78]. However, gadolinium is raising safety concerns, especially due to its potential leaching in the absence of chelates [79].

Therefore, the behavior of lanthanide metals in the body through their metabolism and cell interactions remains to be elucidated to fully ensure the safety of UCNPs [80], while less toxic rare-earth elements, such yttrium, should be preferred to gadolinium [81].

### 3.3. Carbon Dots

Carbon dots (CD) are a new kind of NP, gaining growing interest since 2004. They are made from carbonated molecules with wide approach either from fragmented bulk material or carbonized soluble substrate. 

Their synthesis mainly implicates calcination and/or solvothermal process, either at high or low temperature in acidic or basic conditions. The production of a graphene core depends on the organic precursor and the synthesis process. 

To significantly enhance the photophysical properties, the carbon particle core can be doped with an inorganic salt such as ZnS before surface functionalization [82]. Similar to QD, some CDs are characterized by high quantum yields and photostability with the advantage to be purely organic [83]. CD-labeled matrigel grafted in mice can be detected in the NIR range with a SBR above 2 [59].

Therefore, low-cost CDs have been rapidly investigated in bioimaging showing high biocompatibility and acceptable fluorescence properties [84,85].

### 3.4. Aggregation-Induced Emission Dyes

Self-quenching is a well-known phenomenon common to many organic dyes such as ICG or fluorescein, which lose their fluorescence efficiency at high concentrations or upon aggregation. By contrast, some organic luminophores emit fluorescence only in the aggregated state. This phenomenon, namely aggregation-induced emission (AIE), takes advantage of high brightness, strong photostability and, as with most classical organic fluorophores, good biocompatibility [86].

However, their high hydrophobicity requires an encapsulation step by adding amphiphilic polymers, such as pluronic F127 in organic solvent such as chloroform or tetrahydrofuran. Solvents are eliminated through evaporation. Then, mixes of AIE luminogen and polymer are resuspended and sonicated to obtain hydrophilic AIE NP.

Intravenously injected AIE NPs accumulate in tumors by the EPR effect, and detection of sub-millimetric peritoneal tumors were achieved with the satisfying SBR of 7.2 [87]. In addition, targeting of AIE NPs can be also proposed. NIR AIE NPs with folic acid as targeting agent display enhanced fluorescence in folate receptor positive MCF7 cells and in subcutaneous tumor-bearing mice [60].

### 3.5. Silica-Encapsulated Dyes

Finally, silica-encapsulated dyes differ from the NPs described above by the protective confinement offered by the silica shell [61,88]. Silica surface confers the advantage to be easily adapted to various imaging modalities and targeting agents. For example, NIR dyes such as cyanine 7 encapsulated in a silica nanoparticle were investigated for sentinel lymph node mapping [88]. They demonstrated enhanced brightness and photostability, which made them interesting probes for long operative times without the need for reinjection or high initial dose.

Cornell dots (C-dots) are ultrasmall (less than 10 nm) core/shell silica NPs, which can fulfill FGS requirements with their remarkable properties. Their synthesis relies on a modified sol-gel process, where cyanine derivative is crosslinked with silica precursor, and react to form a fluorescent core. A silica shell is then added to form a core shell structure.

To date, the C-dots are the only type of silica nanoparticles to have reached clinical trial phase I. First, after encapsulation of cyanine 5 NIR-fluorescent dye, the emission of the dye remains unchanged while both photostability and brightness were greatly increased, leading to the enhancement of SBR [62]. Second, C-dots were designed to avoid the major drawbacks of the QDs as with NPs: bio-accumulation and induced toxicity [89]. Ultrasmall, to target the renal excretion windows, C-dots can be renally excreted intact from animals [61] and humans [63]. Besides the reduction of the cytotoxic risk, this fast excretion offers better imagery by rapid reduction of the remnant probe background. Third, these NIR-fluorescent NPs possess all advantages of the versatile surface chemistry of silica NPs. Appropriate coating, for example with PEG, can be easily applied and associated with a targeting agent such as cRGDY, which target αvβ_3_-integrins and with another imaging agent such as the smallest radioactive iodine covalently linked to the cRGDY moiety. It is acknowledged that integrins play a key role during the whole metastatic process of ovarian cancer [64]. Therefore, αvβ_3_-integrin appears to be an appropriate target for this type of cancer [90], although integrin expression may vary between patients [91]. For this purpose, Philipps et al. (2014) performed PET imaging using such a construction on patients with various types of cancer, allowing the detection of different integrin-positive lesions, from the liver to the pituitary gland, proving that intravenously injected C-dots can cross blood–brain barrier [63]. Therefore, an FGS probe should be available with different targeting to fulfill individual patient needs.

Currently, C-dots have entered early phase I clinical trials (NCT02106598) for Image-Guided Intraoperative Sentinel Lymph Node Mapping in Head and Neck Melanoma, Breast and Gynecologic Malignancies. Thus, C-dots are the first NPs to enter clinical trials for FGS. Completion of the study is expected in 2018.

Independent of fluorescence imaging, another property of C-dots was recently highlighted by showing the death of nutrient-deprived cancer cells exposed to C-dots by ferroptosis [65]. This property was confirmed in vivo in two different tumor models, thus conferring unexpected theranostic properties to C-dots. 

Finally, C-dots seem able to fill all the criteria required in clinical bioimaging: high brightness, versatility, multimodality and targeting possibilities. In addition, C-dots are safe due to the fast clearance of the unbounded probe and theranostic properties. However, voluntarily made ultrasmall to target the renal clearance window, the few-nanometer size of the C-dot combined with a large-size antibody or a Super Paramagnetic Iron Oxide Nanoparticle (SPION) could hamper renal elimination, resulting in a decrease in safety. 

## 4. Toward the Short-Wave Infrared

Another possibility to considerably improve the fluorescence imaging efficiency is to increase the infrared wavelength emission of the probe. Short-Wave InfraRed (SWIR) between 1000 and 2500 nm is gaining increasing attention in biological application, especially because of biological chromophore absorption and tissue scattering that are reduced in the 1000–1300 nm “transparent” window. Computational and in vitro simulation have long predicted that SWIR fluorescence could greatly outperform NIR fluorescence imaging, but SWIR imaging cameras have not been made widely available until recently [92]. Therefore, FGS devices were mainly focused on the NIR window and the development of FDA-approved NIR excitation sources [32]. 

SWIR cameras based on InGaAs sensor offer broad imaging facilities in the SWIR range [93]. Currently they are widely available in many fields of investigations including medical [94] and clinical predevelopment devices [95]. Moreover, the prediction of SWIR dye superiority as compared to NIR ones was confirmed by bioimaging [96,97]. While NIR provides a penetration depth of one or two millimeters, SWIR easily pushes the depth limit to five millimeters [98] making SWIR promising, especially for peritoneal cytoreduction.

**Figure 2:**
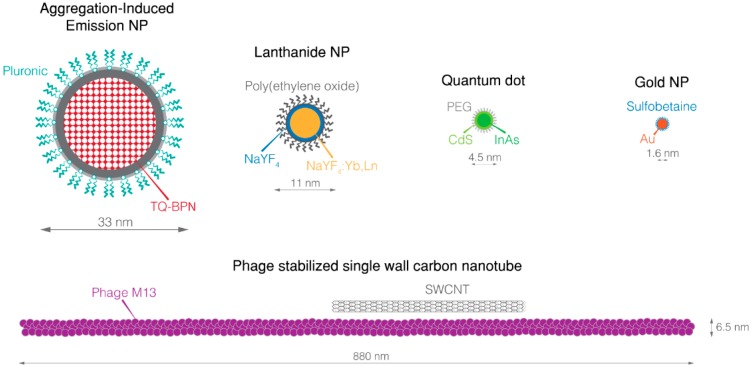
Overview of SWIR nanoparticles. NP, nanoparticle; PEG, poly(ethyleneglycol); SWCNT, Single-walled carbon nanotube; TQ-BNP, *N*,*N*′-((6,7-diphenyl-[1,2,5]thiadiazolo[3,4-g]quinoxaline-4,9-diyl)bis(4,1-phenylene))bis(*N*-phenylnaphthalen-1-amine).

However, SWIR dyes remain scarce and not efficient enough. The state-of-the-art dye is the IR-1050 from Nirmidas biotech (Palo Alto, CA, USA) which has several drawbacks [99]. Despite encouraging characteristics and safe renal excretion, IR-1050 displays very low SWIR fluorescence as compared to NIR dyes [100]. As with all organic dyes, IR-1050 is subjected to photobleaching and the attempt to associate it with a targeting agent and/or another imaging modality is similar to trying to square the circle. SWIR-emitting organic dyes are currently under development but their fluorescence quantum yield does not exceed a few percent [101]. Therefore, SWIR-emitting inorganic NPs (Figure 2) appear as interesting alternatives (Table 2). 

### 4.1. SWIR QD

Similarly to NIR QDs, the main interest of SWIR QD as compared to other NPs is their outstanding photophysical properties, especially for Ag_2_Se QD and InAs QD, resulting in a better signal and higher SBR [96,102,103]. Similar to NIR-emitting QDs, it will be necessary to develop bright SWIR QDs devoid of heavy metals and carefully characterize their in vivo degradation and their potential toxicity. 

### 4.2. Lanthanide Nanoparticles

Next, by their high QY, SWIR lanthanide NPs gained attention for bioimaging. Indeed, several lanthanide NPs have been developed to be absorbed in NIR and to emit in SWIR by the doping of sodium yttrium or gadolinium tetrafluoride nanocrystals, made using a solvo- or hydrothermal process, with different rare-earth elements. These NPs, encapsulated in a 100 nm hydrodynamic diameter albumin shell, were shown to be confined in the peritoneum at least 12 h after intraperitoneal injection [104], while another group reported more than 48 h confinement for similar NPs [108]. Other SWIR fluorescent lanthanide NPs, encapsulated in 100 nm polymeric shell, led to the detection of tumor deposits up to 72 h after intraperitoneal injection in a murine ovarian peritoneal carcinomatosis model. In this case, the process by which NPs remains for a long time in the cavity is still unknown [109]. The undeniable advantage of lanthanide NPs is their relative safety, mainly because of the absence of heavy metal in their composition. For example, microparticles of radioactive yttrium were approved as radiotherapeutic agents for liver malignancies [110]. On the other hand, leaching of Gd ions from NaGdF4 may present severe long-term toxicity issues. If photophysical properties remain a concern for lanthanide nanoparticles, some coatings, such as silica or NaGdF4, have greatly improved it, at least in vitro for now [111].

### 4.3. Gold Nanoparticles

By reducing chloroauric acid in the presence of lipoic acid sulfobetaine, Chen et al. obtained SWIR fluorescent gold NPs with satisfying biocompatibility. Indeed, these NPs exhibited renal excretion and fast clearance in healthy mice, with fluorescent-observable excretion from circulation to kidney, despite a weak fluorescence. Further studies in animal tumor models could provide more interesting results [105].

### 4.4. Carbon Nanoparticle

Single-walled carbon nanotubes (SWCNT) are acknowledged for their SWIR emission under NIR excitation. Several synthesis methods exist to produce SWCNT, such as arc discharge, laser ablation and several chemical vapor deposition processes, which are far more productive. The nanoparticle reviewed below is a commercially available SWCNT made using a specific chemical vapor deposition process named high-pressure carbon monoxide method. In this process, carbon monoxide, which acts as carbon source, and iron carbon monoxide catalyst are continuously injected at high temperature, forming high-quality SWCNT.

Despite the low fluorescence of SWCNT, they have pure carbon composition which do not raise many concerns about QD heavy metal content [112]. Their potential toxicity can be adjusted by using appropriate length and coating. Additionally, depending on their design, they can be safely urinarily excreted or biodegraded [113].

Investigated in a murine model of ovarian peritoneal carcinomatosis, SWCNT displayed high imaging capacity upon 808 nm laser excitation. The SBR was superior to 5 in vivo, and up to 100 ex vivo [106]. Compared to unguided surgery, SWCNT guided surgery offered a significantly better detection, with ten times more sub-millimetric tumors excised through fluorescence guidance.

### 4.5. SWIR Fluorescent Organic Nanoparticles

Several AIE luminogens have a NIR absorption spectrum and display both NIR and weak SWIR fluorescence. Encapsulated in an organic shell such as the pluronic one, these NPs show extended photostability and allow clear visualization of tiny vessels in tissue below 0.8 mm in depth with an SBR higher than 30. They provide suitable properties to detect highly vascularized tumors when used to observe the EPR effect in the subcutaneous murine tumor model [107].

These NPs have the greatest advantage of nontoxic composition, and no adverse effects were observed after intravenous injection. However, their low photophysical properties in the SWIR region is a limiting factor for in-depth detection. Another disadvantage is related to the alteration of fluorescence and photostability upon the addition of a targeting moiety or/and another imaging agent to the NP [114]. 

## 5. NP Safety: A Major Concern

NPs are the main attractive newcomer in the field of pharmaceutics and biomedicine over the last decade. However, their exceptional properties raise major concern about safety. NP design significantly affects toxicity as well as targeting ability and biodistribution behavior of NP in vivo [115]. NPs can rely on their nanosize to cross biological barriers and to reach the most sensitive organs [116], ultrasmall NP (few nanometers) are easily endocyted, where then can disrupt cell biochemistry [117]. Therefore, these NPs are presumed more toxic than their larger counterparts [118]. It was proven that a retention for a long period in many organs such as lung and liver can be harmful, thus careful surface functionalization and passivation of NP is important for safe clinical application [119,120,121].

### 5.1. Urinary Excretion Is Mainly a Matter of Size

Safe application in clinics suggests total excretion of drugs from the organism. Therefore the main criteria to use these NPs clinically are an ultrasmall size (<5.5 nm) and/or an encapsulation in a biocompatible material likely to promote the excretion renally [122]. Additionally, NPs for FGS require appropriate photophysical properties. Considering that, inorganic NPs, especially QDs, are potent agents which could provide excellent imaging capacities if they can be excreted. Ultrasmall QDs were designed to avoid Kupfer cell endocytosis and to reach the bladder through renal filtration. Choi et al. tested cystein-coated Cd/Se QD of varying sizes and different emission wavelengths. Only the smallest QDs (less than 6 nm) were clearly removed by the renal pathway and were collected in the bladder, whereas the largest ones accumulated mainly in the liver, lungs and spleen [122]. The authors concluded that NPs should have a hydrodynamic diameter below 5.5 nm to achieve complete elimination from the body. However, the excretion rate of ultrasmall NPs is more complex than it seems. In fact, a recent study reported some differences between NPs made with varying amounts of gold atoms. Consistently, 1.7 nm NPs (201 gold atoms) are faster renally removed than 2.5 nm ones, while, surprisingly, lower excretion rate was measured with smallest NPs made of less than 20 atoms of gold. Authors also highlighted the role of the renal glycocalyx which acts like a chromatography filtration gel that allow larger NPs to pass rapidly through [123]. Therefore, it can be assumed that the ideal diameter for a NP is comprised between 1.7 nm and 6 nm to target renal excretion and to obtain an effective clearance. The clinical use of NIR Cd-based QD appears unlikely since many of them did not fit size condition.

Another approach to facilitate the renal excretion is using biocompatible coating such as silica-phosphonate for Cd-based QD. From 11.5 nm hydrodynamic diameter core, Ma et al. produced core/shell QD/silica-phosphonate NPs with a diameter of almost 30 nm. Despite its relatively large hydrodynamic diameter, the NP was mainly urinarily excreted after intravenous injection. In addition, the silica-phosphonate coating produced extended circulation time in blood to decrease liver accumulation [124].

Finally, the design of biodegradable NPs is still under investigation to reach excretion of NP after intravenous injection [125,126]. This approach can be easily applied to silica NP, AIE NP, carbon dot and SWCNT; however, its utilization for NP made of heavy metals or rare-earth elements is limited. In this context, biodegradable and heavy metal-free QDs constitute attractive alternatives. Among them, NIR silicon QDs [127] are small enough (<5 nm) [128], highly biocompatible and are able to be endocytosed by cancerous cells [129]. No toxicity was detected both in mice and monkey models even at high dose of QD (200 mg/kg). The size of silicon QDs varied from 4 to 11 nm, so they are rapidly accumulated both in the liver (the largest fraction) and in the bladder (the smallest fraction). Three months post-injection, high silica content was found in the liver and the spleen due to the retention of the largest QDs in these organs. Consistently, liver damage was histopathologically observed in mice but not in monkeys, suggesting the influence of the anatomical scale between these models exposed to identical amount of silicon QDs [130].

### 5.2. Rethinking of the Injection Route

Among the drawbacks in toxicity, fluorescent contamination by unbounded dye also must be considered for FGS with NPs. Evidently the biodistribution of fluorescent dye significantly depends on the injection route. For example, a large part of intravenously injected organic dye such as ICG is excreted through the liver and the intestine for a long time. The remaining part of the probe in these organs produces fluorescence contamination which can overshadow the fluorescence emitted by the cancerous tissue, decreasing SBR. Therefore, the renal excretion route is preferable for intravenously injected FGS drugs. A seen above, NPs require small size (less than 6 nm) [123] and probably a specific shape to be renally excreted [131]. This has already been shown clinically with the example of Cornell dots. However, the size of nanoparticle can be adjusted only for the limited numbers of nanomaterials. Thus, whenever possible, systemic exposition should be avoided as the simplest solution. 

Additionally, intravenously injected probes demonstrated limited efficiency in the case of sub-millimetric-sized and/or small tumors which are not yet vascularized [132]. For these kind of tumors, the advantages of injection had been proved for organic NIR dyes. After intravenous injection, fluorescent contamination of many organs was observed. The appropriate SBR was displayed only for tumors larger than 5 mm. On the contrary, intraperitoneally injected dye allows detection of small tumors with reduced fluorescent contamination [133]. According to that, the use of the intraperitoneal injection route prevents systemic exposition and fluorescent contamination and provides an opportunity to safely use nanoparticle in clinics for peritoneal carcinomatosis FGS.

Biodistribution of NPs injected by the intraperitoneal route has been recently investigated in several studies reporting promising results. The intraperitoneal injection of QDs facilitated the dissection of peritoneal lymph nodes during the cytoreduction in rats due to rapid lymphatic drainage of the QDs [134]. Kato et al. (2010) monitored the biodistribution of intraperitoneally injected captopril QD by means of mass spectrometry [135]. The authors observed significant difference between QDs injected intraperitoneally and intravenously: only 2.5% of the initial dose of QDs in the liver, 1.5% in the spleen and almost 8% in the bloodstream, and almost 85% of the QDs were not detected in organs and seemed to remain confined to the peritoneal cavity six hours after intraperitoneal injection. Finally, injection of QD by intraperitoneal route showed appropriate toxicity. Adverse effects of mercaptopropionic acid coated QD were observed in mice only after 15 days of repeated intraperitoneal injection of 10 mg/kg [136]. QD induced mild toxicities in liver and lung, which they were detected by fluorescence microscopy. 

Obviously intraperitoneally injected NPs can passively accumulate in ovarian peritoneal tumors e.g., lanthanide NPs [109]. However, the majority of FGS drugs possess low tumor selectivity which can be improved by using active targeting molecules. For example, SWCNTs functionalized with the secreted protein acidic and cysteine rich (SPARC)-binding peptide, actively targeting tumors with an SBR up to 5, remaining confined to peritoneal cavity at least for 24 h [106]. Indirect targeting can be also used with success in murine peritoneal carcinomatosis by injecting the peptide iRGD first, to permeate the tumor tissue before injecting NIR QDs. Then, unbounded QDs were bleached by using an etchant, and this step of the procedure allowed the detection of QD-labeled tumors [57]. To increase SBR, a peritoneal washing procedure could also be applied to any type of NPs. At the same time, the “washing” of a peritoneal cavity by etchant could enhance the safety of QDs by removing heavy metals from the organism, for example, in the case of ZnSeHg QDs [57].

## 6. Conclusions

Ovarian carcinomatosis FGS requires safe drugs, which selectively accumulate in the malignant tissue and provide high SBR for complete cytoreduction. NIR-fluorescent NPs possess all necessary characteristics to be potent FGS probes. To date, Cornell dots are the safest type of NIR NP, which is already in clinical trial phase I. The special design of Cornell dots results in rapid excretion renally following intravenous injection but were never applied for peritoneal carcinomatosis. By contrast, CD, UCNP and QDs were already studied using the intraperitoneal injection route, which is suggested to be the most potent for the detection of ovarian metastases. Obviously, the intraperitoneal route avoids systemic exposition, improving NP safety and providing the opportunity to use active targeting molecules to enhance selectivity and SBR of NPs. Another way to improve SBR is to use SWIR fluorescent NP, which demonstrated extended photostability and provided visualization of tiny vessels below 0.8 mm in tissue depth with an SBR higher than 30. Finally, NPs can be associated with other imaging agents in multimodal approaches to achieve pre-operative whole-body imaging, and precise tumor detection to complete cytoreduction. 

Currently, NIR cameras and lasers are already FDA-approved for FGS. SWIR cameras have also become available on the market and are expected to be approved for medical use. In fact, the application of FGS in NIR and SWIR is limited by the number of FDA-approved dyes (ICG and methylene blue), therefore the investigation of NP-based FGS probes is of great interest.

## Figures and Tables

**Table 1:** Fluorescent near-Infrared Dye and their characteristics.

Spectra	Near-Infrared						
Name	ICG	OTL-38	Quantum Dots	UCNP	Carbon Dot	AIE NP	Cornell Dots
Component	C_43_H_47_N_2_NaO_6_S_2_	C_61_H_63_N_9_O_17_S_4_/4Na	CuInSe/ZnS(Mn) ZnSeHg	Yb, Tm, Er doped NaYF_4_ nanocrystal/NaLuF_4_ shell	Graphite core	Organic core	Cyanine 5 core and silica shell
Size (nm)	−	−	9.0 (CuInSe/ZnS(Mn) 6.6 (ZnSeHg)	30	11	46	5.5
Coating	−	−	PEG	PEG	PEG	PEG	PEG
Targeting	−	Folate	iRGD	−	−	Folate	cRGD
Excretion	Hepatobiliary	Hepatobiliary	−	−	−	Hepatobiliary	Renal
Multimodality	−	−	MRI (Mn)	−	−	−	PET (^124^I)
Photostability	Low	Low	High	High	High	High	High
Excitation (nm)	805	774	690 (CuInSe) 785 (ZnSeHg)	980 (multiphotonic)	633	635	650
Emission peak (nm)	835	794	685(CuInSe/ZnS(Mn)) >800 (ZnSeHg)	800	>710	810–815	670
SBR of *i.p.* tumor	2 ± 1	4.4	12	>5	−	7.2	−
Results in vivo	−	−	−	Passive accumulation in peritoneal tumors following *i.p.* injection	SBR ≈ 2 in subcutaneously injected matrigel	Allow the detection of sub-millimetric peritoneal tumors	−
Clinical	Low specificity	Improved cytoreduction	−	−	−	−	Preferential uptake of Cornell dots at the site of the disease, in vivo stability and safety
Reference	[47,48,49]	[50]	[56,57]	[58]	[59]	[60]	[61,62,63,64,65]

Abbreviations: AIE: aggregation-induced emission; ICG: indocyanine green; *i.p.*: intraperitoneal; MRI: molecular resonance imaging; NP: nanoparticle; OTL: on target laboratories incorporated (West Lafayette, USA); PEG: poly(ethyleneglycol); PET: positron emission tomography; SBR: signal-to-background ratio; UCNP: up-converting nanoparticle.

**Table 2:** Short-Wave Infrared fluorescent nanoparticles and their characteristics.

Spectra	Short-Wave Infrared						
Name	IR-1050	ICG	Quantum Dot	Lanthanide NP	Gold NP	Phage Stabilized SWCNT	AIE NP
Component	C_41_H_40_BC_l3_F_4_N_2_	C_43_H_47_N_2_NaO_6_S_2_	Ag2S InAs	NaYF_4_ Yb:Ln core doped with rare-earth NaYF_4_ shell	Gold	Pure carbon nanotube	Organic core
Size (nm)	−	−	3.0–4.0 (Ag_2_S) 4.5 (InAs)	9.0–11	1.6	880 × 6.5 *	33
Coating	−	−	PEG	Polymeric coating by poly(ethylene oxide)	Lipoic acid-based sulfobetaine	Phage M13	Pluronic
Targeting	−	−	−	Folate	−	SPARC-Binding peptide	−
Excretion	Hepatobiliary	Hepatobiliary	Hepatobiliary (Ag_2_S)	−	Renal	−	−
Multimodality	−	−	−	−	−	−	−
Photostability	Low	Low	High	High	High	High	High
Excitation (nm)	790	805	808	980	808	808	630
Emission peak (nm)	1050	835	1125 (Ag_2_S) 1080–1330 (InAs)	1185 (Ho doped) 1310 (Pr doped) 1475 (Tm doped) 1525 (Er doped)	800–1400	1000 – 1300	808
SBR of *i.p.* tumor	−	−	14 (Ag_2_Se)	>3	−	8	−
Results in vivo	−	−	*i.v.* injected Ag_2_S QDs passively accumulate in subcutaneous. murine tumor with a ratio of 10% ID/g tumors	*i.p.* injected lanthanide NPs accumulate, with or without targeting, in *i.p.* tumors from ovarian cancer OVCAR8 cell line	−	Effective imaging of peritoneal tumors after *i.p.* injection, with higher resection rate, especially for sub-millimetric nodules	SBR is 33 at the depth of 150 µm in mouse brain vasculature following *i.v.* injection
Clinical	−	−	−	−	−	−	−
Reference	[100]	[100]	[93,102,103]	[104]	[105]	[106]	[107]

*—the size of carbon nanotubes is presents as height × diameter. Abbreviations: AIE: aggregation-induced emission; ICG: indocyanine green; ID: injected dose; *i.p.*: intraperitoneal; *i.v.*: intravenously; NP: nanoparticle; PEG: Poly(ethyleneglycol); QD: quantum dot; SBR: signal-to-background ratio; SPARC: secreted protein acidic rich in cysteine; SWCNT: single-walled carbon nanotube.
